# pBLAM1-x: standardized transposon tools for high-throughput screening

**DOI:** 10.1093/synbio/ysad012

**Published:** 2023-06-21

**Authors:** Lorea Alejaldre, Ana-Mariya Anhel, Ángel Goñi-Moreno

**Affiliations:** Centro de Biotecnología y Genómica de Plantas, Universidad Politécnica de Madrid (UPM)—Instituto Nacional de Investigación y Tecnología Agraria y Alimentaria (INIA/CSIC), Madrid, Spain; Centro de Biotecnología y Genómica de Plantas, Universidad Politécnica de Madrid (UPM)—Instituto Nacional de Investigación y Tecnología Agraria y Alimentaria (INIA/CSIC), Madrid, Spain; Centro de Biotecnología y Genómica de Plantas, Universidad Politécnica de Madrid (UPM)—Instituto Nacional de Investigación y Tecnología Agraria y Alimentaria (INIA/CSIC), Madrid, Spain

**Keywords:** Genetic tool, Chromosomal insertions, Transposon, Plasmid, Standard format

## Abstract

The engineering of pre-defined functions in living cells requires increasingly accurate tools as synthetic biology efforts become more ambitious. Moreover, the characterization of the phenotypic performance of genetic constructs demands meticulous measurements and extensive data acquisition for the sake of feeding mathematical models and matching predictions along the design-build-test lifecycle. Here, we developed a genetic tool that eases high-throughput transposon insertion sequencing (TnSeq): the pBLAM1-x plasmid vectors carrying the Himar1 Mariner transposase system. These plasmids were derived from the mini-Tn5 transposon vector pBAMD1-2 and built following modular criteria of the Standard European Vector Architecture (SEVA) format. To showcase their function, we analyzed sequencing results of 60 clones of the soil bacterium *Pseudomonas putida* KT2440. The new pBLAM1-x tool has already been included in the latest SEVA database release, and here we describe its performance using laboratory automation workflows.

**Graphical Abstract** 
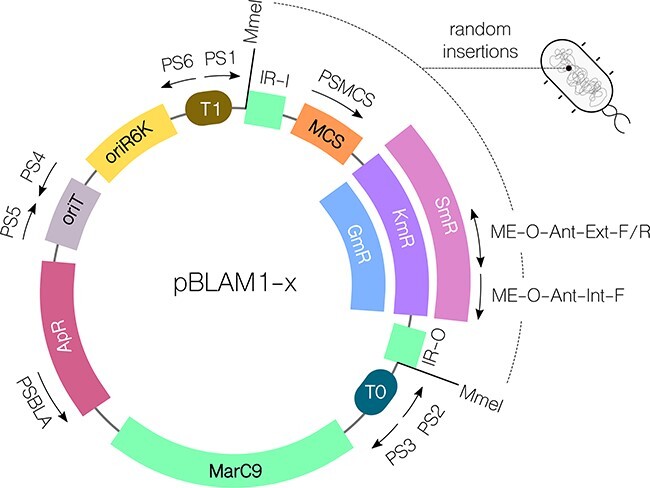

## 1. Introduction

Genome engineering is a powerful alternative to the use of plasmid vectors in microbial synthetic biology to create stable insertions of genetic circuits or metabolic routes and to prevent unwanted phenotypic variability (1–5). Nowadays, there are several approaches to genomic insertions, such as the use of targeted or random insertion transposases (6–8), modified bacteriophages (9), homologous recombination (10) or CRISPR-Cas (11, 12). Among these approaches, random insertion of genes with transposases is a straightforward method to produce genetic diversity, develop new strains or to identify essential regions within the genome (13–16). The use of random transposase insertions has allowed researchers to pin-point genomic locations important for cell survival (14), to create minimal genomes (15) or to perform strain development (17, 18). Its combination with high-throughput sequencing methods (TnSeq) has accelerated the exploration of genomic locations that allow stable genetic insertions or higher expression yields (7, 13, 14, 19). Toward this goal, mariner transposases are the preferred transposition system for random integration due to the simplification in library creation and bioinformatic analysis, increase in sequencing depth and lower biases for fitness calculations (7, 20–22).

A variety of transposase systems are available, allowing alternatives depending on whether the goal is targeted sequencing methods, transposition efficiency or a more comprehensive exploration of the genomic sequence space. The two most commonly used transposases for random genomic insertions into bacteria are the Tn5 and mariner transposase systems (13, 23). Although these transposases are random, they exhibit specific sequence preferences and integration biases as a result. Tn5 inserts more frequently into GC-rich genomic regions (6, 24), whereas mariner transposases target TA sites with a slight sequence bias in the flanking nucleotides (24). Mariner transposase insertions are often considered to be less biased and are therefore frequently used for evaluating gene essentiality (19, 23, 24). The biases observed in transposase experiments are largely dependent on the GC content of the target genome and thus varies between species (24, 25). Other factors such as DNA bendability, which is hard to anticipate, can also bias transposon insertions (26). Of the currently available random transposase systems, only the Tn5 transposase system has been included in a broad-host-range plasmid following Standard European Vector Assembly (SEVA) standardization guidelines (6, 27–30).

Standardization of molecular biology tools provides the means to increase reproducibility, protocol exchange and optimized laboratory automation workflows (31–33). In this work, we sought to increase the available genomic integration toolbox for bacteria by creating a broad-host-range plasmid set that includes the hyperactive form of the Himar1 mariner transposase (MarC9) (26) and that is compatible with SEVA standardization guidelines. Hyperactive forms of transposases ensure a higher transposition and are better suited for *in vivo* work (26). In addition, Himar1 mariner transposase, isolated from the hornfly *Haematobia irritans* (26), has been successfully used in several gram-negative bacterial species such as *Escherichia coli* (26), *Pseudomonas putida* (34), *Pseudomonas aeruginosa* (35), *Aggregatibacter actinomycetemcomitans* (36) *Caulobacter crescentus* (24)*, Rhizobium leguminosarum*^24^) *and Vibrio cholerae* (24), gram-positive such as *Bacillus subitilis and Streptococcus pneumoniae* (7) as well as several Mycobacteria (24, 37).

The new vector set, termed pBLAM1-x (*Born to Life Again mini-Mariner transposon*) is available with three different antibiotic resistances to facilitate sequential insertions (pBLAM1-2: Kanamycin; pBLAM1-4: Streptomycin; and pBLAM1-6: Gentamycin). It has been included in the SEVA 4.0 update (30) and is available through the SEVA database. We showcase their use for genomic insertions in the microbial chassis *P. putida* KT2440, as well as the genotypic characterization via open-source automation. The compatibility of the automation workflow presented in this work with other SEVA systems eases its implementation in laboratories that already follow SEVA standardization guidelines. The use of the pBLAM1-x set to facilitate TnSeq is not addressed in this work as the use of mariner transposases in *P. putida* and other pseudomonads (22, 34, 35, 38), as well as other gram-negative, gram-positive and mycobacterial systems has been previously validated (24, 39. However, this feature remains an advantage to accelerate genotype-to-phenotype relationships of inserted synthetic genetic circuits and design-build-test cycles.

Overall, our results demonstrate the use of this vector set to introduce genomic modifications. This addition to the synthetic biology toolkit facilitates protocol standardization as well as massive parallel sequencing of insertion libraries.

## 2. Materials and methods

### 2.1. Reagents

Plasmids were obtained using the E.Z.N.A. plasmid DNA mini kit II (Omega Bio-Tek) from bacterial cultures (Table S1), PCR products were purified using the DNA clean and concentrator kit-5 (Zymo Research). Oligonucleotides were synthesized by Integrated DNA Technologies (IDT-DNA) (Tables S2–S4). Restriction enzyme DpnI was obtained from New England Biolabs; Phusion polymerase, Phire Green Hot Start II PCR Master mix and Phire Hot Start II PCR Master mix were obtained from ThermoScientific.

### 2.2. pBLAM1-x set construction

The pBLAM1-x set has been constructed sequentially. First, the hyperactive Tn5 transposase *tnpA* gene in pBAMD1-2 was replaced with the hyperactive mariner transposase gene *marC9* through modified restriction free cloning (40) adding a final step after DpnI digestion (‘magic-touch PCR’ modification: 98°C 38 s, 64°C 2 min, 72°C 5 min, 98°C 8 s, 58°C 2 min, 72°C 5 min, 98°C 8 s, 50°C 2 min, 72°C 5 min, 72°C 5 min). Oligonucleotides F1-Mariner-pBAMD1-2 and R1-Mariner-pBAMD1-2 (Table S2) with sequence homology to *marC9* gene and the sequence surrounding *tnpA* gene in pBAMD1-2 were used to amplify *marC9* from pMarC9-R6K (Addgene plasmid #89477) (41). The purified PCR product was then used in a second PCR reaction using pBAMD1-2 as template to replace the Tn5 transposase gene for *marC9*. Primer design and PCR conditions were those recommended in the restriction free cloning tool (42). The second PCR product was digested for 2 h at 37°C with DpnI (New England Biolabs) to eliminate the template pBAMD1-2, followed by a ‘magic-touch PCR’ step and transformed into competent *E. coli* pir^+^ cells. Upon verification of the correct replacement of *tnpA* for *marC9*, the mosaic elements ME-I and ME-O recognized by TnpA were replaced by inverted repeats termed IR-I and IR-O, which contain MmeI restriction sites, recognized by MarC9 transposase. This step was done through a single-step site-directed mutagenesis protocol (43) using primers F1-IR-PacI, R1-IR-SpeI, F1-IR-SpeI and R1-IR-PacI (Table S2). Primer pairs F1-IR-PacI/R1-IR-SpeI and F1-IR-SpeI/R1-IR-PacI were used in separate reactions to substitute ME-O and ME-I in pBAMD1-2 containing *marC9* for 16 cycles using an annealing temperature of 50°C for 30 s, 30 s of extension at 72°C and standard conditions for Phusion polymerase (Thermo Scientific) in a 25 µL volume. Then, reactions were mixed to a total of 50 µL, 0.5 µL of polymerase was added and the reaction continued for 12 additional cycles modifying the annealing temperature to 55°C. The reaction was digested with DpnI, subjected to ‘magic-touch PCR’ and transformed. The obtained plasmid was termed pBAMD1-2-Mariner-IR-PacI/SpeI. Lastly, a SacI site in *marC9* was edited to allow for traditional cloning using the standardized MCS. Whole-plasmid site-directed mutagenesis (44) was done with partially overlapping primers F-QC-SacI-MarC9 and R-QC-SacI-MarC9 (Table S2). The PCR reaction was run following the standard conditions for Phusion polymerase using 10 ng of pBAMD1-2-Mariner-IR-PacI/SpeI template in a 50 µL reaction for 12 cycles, melting temperature of 55°C and an extension time of 1 min/kb. The reaction was subjected to the same processing as in the previous cloning steps prior to transformation. This last modification yielded the final plasmid pBLAM1-2.

Versions resistant to streptomycin and gentamicin were created yielding pBLAM1-4 and pBLAM1-6 vectors, respectively. Analogous to the gene transposase replacement, the kanamycin resistance gene present in the cargo module was replaced through modified restriction free cloning (40). Streptomycin and gentamicin resistance genes (smR and kanR) were amplified using primers F1-pBAMD1-2-SmR and R1-pBAMD1-2-SmR (Table S2) from pBAMD1-4 (6) and F1-pBAMD1-2-GmR and R1-pBAMD1-2-GmR (Table S2) from pBAMD1-6 (6). PCR reactions conditions were those suggested by the restriction free cloning tool (42) and subsequent processing that of previous cloning steps.

**Figure 1.**
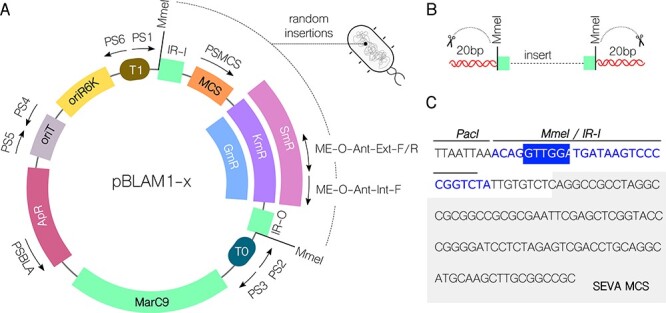
Plasmid map and features of pBLAM1-x mini-mariner vectors. (A) Plasmid map of pBLAM1-x showing PS1-PS6 SEVA primer sites and PSMCS, ME-O-Ant-Ext-F and Int-F primers common to SEVA-Sib pBAMD1-x vector set. Multiple Cloning Site (MCS) is shown in orange, Inverted Repeat (IR) regions and hyper-active Himar Mariner marC9 transposase gene are shown in green, antibiotic resistance to kanamycin (KmR), streptomycin (SmR) and gentamicin (GmR) are shown in purple, magenta and blue respectively, in green, backbone ampicillin resistance (ApR) gene in light burgundy, origin of transfer (oriT) in gray, origin of replication R6K (oriR6K) in yellow and T1 and T0 terminator sequences in brown and teal colors respectively. Type IIs MmeI restriction sites surrounding the cargo in IR repetitive sequences recognized by MarC9 are also shown. (B) Schematic representation of cleavage with Type IIS MmeI restriction enzyme of inserted cargo in the genome to create libraries for TnSeq. (C) Nucleotide sequence from IR-I sequence recognized by MarC9 transposase to the MCS. MmeI restriction site within the IR-I sequence is highlighted in blue. MCS sequence equivalent to SEVA is highlighted in gray.

The intermediate and resulting plasmids pBLAM1-2, pBLAM1-4 and pBLAM1-6 (Figure 1) were verified through Sanger-sequencing by Macrogen-Europe using SEVA oligonucleotides PS1-PS6 (28) and PSBLA (Table S3).

### 2.3. Conjugation into *Pseudomonas putida* KT2440

The pBLAM1-x set were used as donor plasmids in triparental matings to integrate their cargo into the genome of *P. putida* KT2440 in a manner similar to that previously reported (6). The bacterial strains used are described in Table S1. Briefly, PIR2 cells carrying either pBLAM1-2, pBLAM1-4 or pBLAM1-6 were mixed with the helper strain HB101/pRK600 and the recipient strain *P. putida* KT2440 in a 1:1:1 ratio based on their OD_600nm_. All cells were washed with 10 mM MgSO_4_ prior to mixing in a 1:1:1 ratio to reduce the amount of antibiotics in the media, which differs for each strain. After mixing, cells were centrifuged at 5000 g/5 min, resuspended in 10–20 µL of 10 mM MgSO_4_ and a single drop was spotted in a Luria-Bertani agar plate. Plates were incubated at 30°C for 5 h. Subsequently, the whole cell-patch was plated without diluting or in a 10^-2^ dilution in a 145 mm round M9-citrate agar plate plus the corresponding cargo antibiotic. Libraries had noticeable colonies by 48 h. Conjugation reactions were carried out for 5 h and in triplicate to minimize the occurrence of repeated sequences due to replication or integration biases.

### 2.4. Library genotyping

Libraries made with the pBLAM1-x set through conjugation into *P. putida* KT2440 were picked into 96-well plates containing M9-citrate plus the cargo antibiotic and subjected to an automated workflow (45) to identify the region of genomic integration of individual variants; 96 clones were picked for libraries generated with pBLAM1-2 and pBLAM1-4 and 52 clones for that of pBLAM1-6. Our automation workflow uses an open-source liquid handler (OT-2, Opentrons, USA) to: (i) inoculate 96-well plates containing Luria-Bertani plus the cargo antibiotic and M9-citrate media plus ampicillin, (ii) perform counter-selection by selecting colonies that do not grow in the presence of ampicillin and grow in the presence of the cargo antibiotic, (iii) prepare glycerol stock plates for each library and a master PCR plate, (iv) do subsequent colony PCRs to detect spurious integration events (6) using primer pairs PS3/PS4 and PS5/PS6 (Table S3) and (v) perform arbitrary PCRs (6, 46). An optional step to verify the correct integration of the cargo module containing the selective antibiotic marker was also carried out using primer pairs PSMCS and ME-O-Km-Ext-R, ME-O-Sm-Ext-R or ME-O-Gm-Ext-R (Table S4). Arbitrary PCRs were done as previously reported (6) by two subsequent PCR amplifications using primer pairs ARB6 and ME-O-Km-Ext-F, ME-O-Sm-Ext-F or ME-O-Gm-Ext-F for a first amplification and ARB2 and ME-O-Km-Int-F, ME-O-Sm-Int-F or ME-O-Gm-Int-F for a second amplification (Table S4). After amplification through arbitrary PCRs, a 96-well plate containing 20 variants from each library was sent to sequencing to Macrogen-Europe with oligonucleotides ME-O-Km-Int-F (pBLAM1-2), ME-O-Sm-Int-F (pBLAM1-4) or ME-O-Gm-Int-F (pBLAM1-6) (Table S4). Sequencing results were aligned and annotated with a python-based script that uses command-line *blastn* and the genome file and annotation file for *P. putida* KT2440 available at https://www.pseudomonas.com. The annotation script is also described in the mentioned automated workflow (45). The location of each insertion in the genome of *P. putida* KT2440 was mapped using the online tool Proksee available at https://proksee.ca.

## 3. Results and discussion

### 3.1. General plasmid features

The standardization of constructs is an overarching goal within the field (31, 32). Here, we present a transposase vector set using guidelines of the Standard European Vector Architecture (SEVA) format (27–30) (Figure 1). The use of the Tn5 transposition system integrated in a plasmid following SEVA criteria has already been validated for the random integration of cargoes in gram-negative bacterial genomes (6, 47). Here, we expand on the available transposition tools compatible with SEVA criteria by constructing a set of vectors with the Himar1 mariner transposase system with proven broad-host applicability in bacteria (24).

The new pBLAM1-x (*Born to Life Again mini-Mariner transposon*) vector set contains the hyperactive Himar1 mariner transposase *marC9*^26^ gene. It derives from the broad-host-range mini-Tn5 vector pBAMD1-2 and contains the same modular features for replication, selection and conjugation (6). These modules can be amplified by the standardized oligonucleotides PS1–PS6 and are flanked by specific restriction sites, thus facilitating the creation of standardized protocols for cloning, characterization and automatization (27–30). In addition, the maintenance of the ampicillin resistance selection marker in the plasmid’s backbone also allows users to create common protocols for screening spurious integration events. To allow the combined insertion of different genes, we have created versions with antibiotic resistances in their cargo to either kanamycin, streptomycin or gentamicin (Figure 1A).

The inserted region is flanked with Type IIS MmeI restriction enzymes to allow for the generation of TnSeq libraries (Figure 1B) and the multiple cloning site (MCS) is SEVA-compatible (Figure 1C).

### 3.2. Evaluation of conjugation efficiency and insertion in *Pseudomonas putida* KT2440 through an automated workflow

The ability of the pBLAM1-x set to integrate into bacterial genomes was assessed in *P. putida* KT2440. The conjugation efficiency of the pBLAM1-x set was shown to be between 10^−3^ and 10^−^6 conjugants/recipient cells (pBLAM1-2 4.1 × 10^−^3, pBLAM1-4 2 × 10^−4^ pBLAM1-4; pBLAM1-6 1.3 × 10^−6^). However, conjugation efficiencies and insertion biases are largely dependent on the host organism (24) and will likely be different for other organisms. The occurrence of spurious integration events and genotyping was done through an automated workflow that exploits common SEVA features (45). This demonstrated that the number of clones growing in the presence of the backbone antibiotic ampicillin is low (6.3% for pBLAM1-2, 6.2% for pBLAM1-4 and 3.8% for pBLAM1-6). The correct integration of the cargo module containing the selective antibiotic marker was verified except for 3 out of 23 colonies of the pBLAM1-4 library. Analysis of the sequencing results of arbitrary PCRs of 20 colonies per library revealed the successful insertion across the genome of *P. putida* KT2440 (Figure S1, Table S5) with some insertional biases. Note that integrations using plasmid pBLAM1-6 had a higher insertional bias, probably due to its lower conjugation efficiency (three orders of magnitude lower than for pBLAM1-2) and/or host-specific interplay. The Himar1 mariner transposase targets TA sites across the genome with some reported sequence biases (24), which in the case of the high GC content (61.6%) genome of *P. putida* KT2440 (48) means that it can still theoretically integrate at ∼10^5 ^TA sites. The Tn5 SEVA-like transposition system shows an insertion bias toward insertion at genomic locations flanked by G/C pairs (6, 24). Therefore, the pBLAM1-x set can serve as a complementary SEVA-like tool to uniformly target the whole genome of *P. putida* KT2440 by employing the same methods to characterize genomic insertions.

The combination of SEVA features with the advantages of Himar1 transposase for random insertion and creation of next-generation sequencing libraries make this plasmid set a relevant tool to accelerate the directed evolution or evaluation of complex genetic circuits in synthetic biology.

## Supplementary Material

ysad012_Supp

## Material Availability Statement

All pBLAM1-x vectors (pBLAM1-2, pBLAM1-4, pBLAM1-6) are available at the SEVA repository https://seva-plasmids.com
