# Supplementary material for: pBLAM1-x: standardized transposon tools for high-throughput screening
Source: Synth Biol (Oxf). 2023 Jun 21;8(1):ysad012. doi: 10.1093/synbio/ysad012 (PMC10306358; doi:10.1093/synbio/ysad012)
Supplement: ysad012_Supp [file ysad012_supp.zip › suppl_data/pBLAM_Supplementary_Material_LA_AGM_revised.docx]

**Supplementary Material**

**for**

**pBLAM1-x: Standardized transposon tools for high-throughput screening**

Lorea Alejaldre^1^, Ana-Mariya Anhel^1^, Ángel Goñi-Moreno^1 *^

^1^ Centro de Biotecnología y Genómica de Plantas, Universidad Politécnica de Madrid (UPM)—Instituto Nacional de Investigación y Tecnología Agraria y Alimentaria (INIA/CSIC), Madrid, Spain

* angel.goni@upm.es

**Figure S1. Distribution of genomic insertions.**


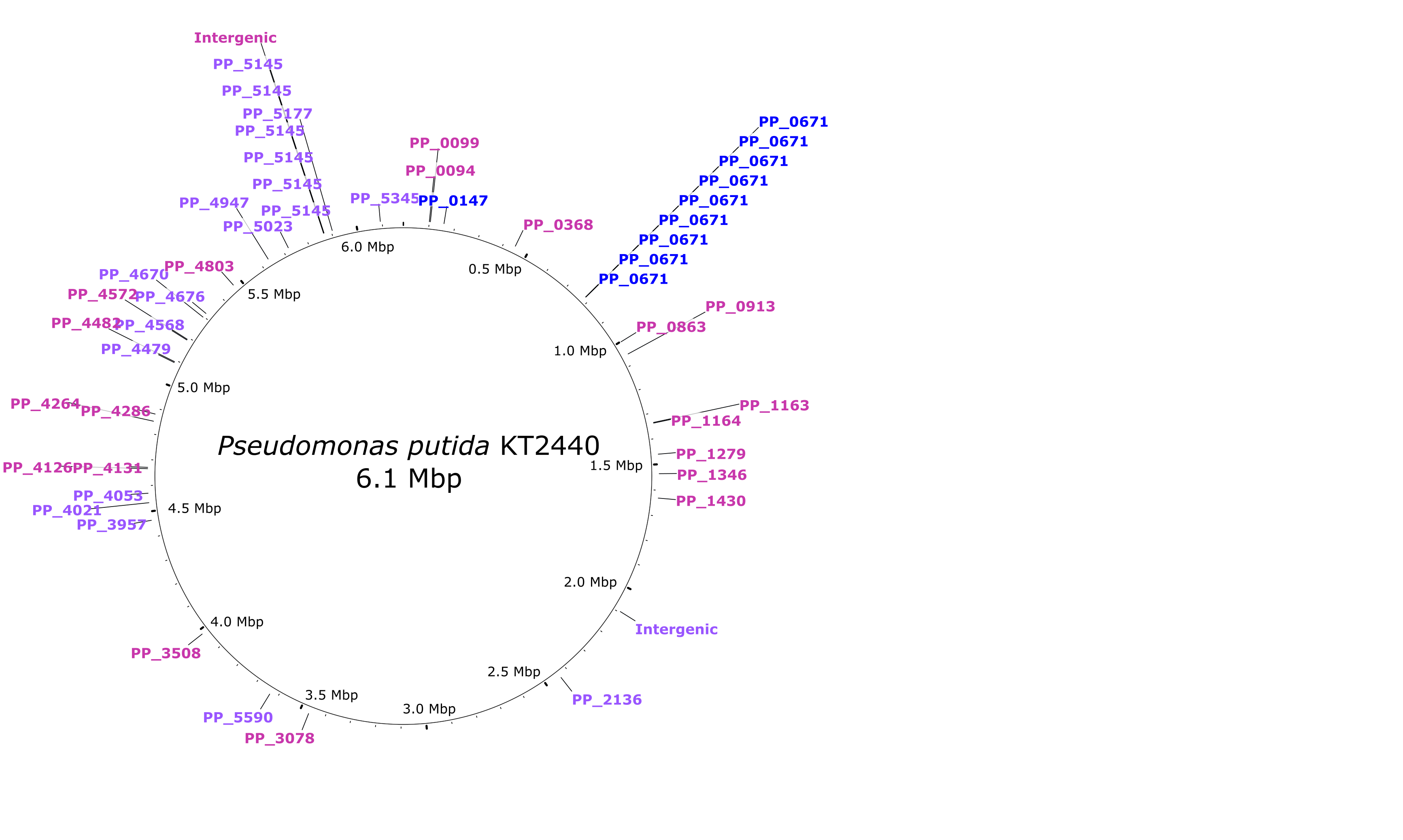


**Figure S1**. Insert location, according to gene locus tag, in the genome of Pseudomonas putida KT2440 of 60 variants created with the pBLAM1-x set coloured according to the delivery plasmid: pBLAM1-2 (purple), pBLAM1-4 (magenta) and pBLAM1-6 (blue). Figure modified from Proksee (proksee.ca) output using annotated sequencing results (S5 Table).

**Table S1. Strains used in this study.**

| **Strain** | **Plasmid** | **Reference** |
| --- | --- | --- |
| *E. coli* PIR2^1^ | - | Invitrogen |
| *E. coli* DH5$\alpha\lambda$ pir^2^ | - | Victor de Lorenzo, CNB-CSIC, Spain |
| *P. putida* KT2440 |  | Victor de Lorenzo, CNB-CSIC, Spain (Martínez-García et al., 2014) |
| *E. coli* HB101 | pRK600 | Victor de Lorenzo, CNB-CSIC, Spain (Martínez-García et al., 2014) |
| *E. coli* DH5$\alpha\lambda$ pir | pBAMD1-2 | Victor de Lorenzo, CNB-CSIC, Spain (Martínez-García et al., 2014) |
| *E. coli* DH5$\alpha\lambda$ pir | pBAMD1-4 | Victor de Lorenzo, CNB-CSIC, Spain (Martínez-García et al., 2014) |
| *E. coli* DH5$\alpha\lambda$ pir | pBAMD1-6 | Victor de Lorenzo, CNB-CSIC, Spain (Martínez-García et al., 2014) |
| *E. coli* PIR1 | pMarC9-R6K | Addgene plasmid #89477(Lee et al., 2019) |
| *E. coli* PIR2 | pBLAM1-2 | This work |
| *E. coli* PIR2 | pBLAM1-4 | This work |
| *E. coli* PIR2 | pBLAM1-6 | This work |

^1^competent cells obtained commercially from Invitrogen

^2^home-made competent cells using CaCl_2_ method (Sambrook & Russell, 2006)

**Table S2. Oligonucleotides used for cloning.** Synthesized by Integrated DNA Technologies (IDT-DNA).

| **Oligonucleotide** | **Sequence** | **Reference** |
| --- | --- | --- |
| F1-Mariner-pBAMD1-2 | GTGACCTCCTAACATGGTAACGTTCAATGGAAAAAAAGGAATTTCGT | This work |
| R1-Mariner-pBAMD1-2 | GGCGGGCTACTAGCGATCGCCGTTATTCAACATAGTTCCCTTCAAGA | This work |
| F1-IR-PacI | TATTTGATGCCTTTAATTAAacaggttggatgataagtccccggtctaTTGTGTCTCAGGCCGCCTAG | This work |
| R1-IR-PacI | tagaccggggacttatcatccaacctgtTTAATTAAAGGCATCAAATAAAACGAAAGGCTCAGTCGAA | This work |
| F1-IR-SpeI | TACAAGCATAAAATCTCTGAtagaccggggacttatcatccaacctgtACTAGTCTTGGACTCCTGTTGATAGATCCAGT | This work |
| R1-IR-SpeI | acaggttggatgataagtccccggtctaTCAGAGATTTTATGCTTGTAAACCGTTTTGTGAAAAAATT | This work |
| F-QC-SacI-MarC9 | TGCCGCGCGAACTCACATTTGACCAAAAACAACG | This work |
| R-QC-SacI-MarC9 | CAAATGTGAGTTCGCGCGGCACCCATTTCG | This work |
| F1-pBAMD1-2-SmR | TGCTTACATAAACAGTAATACAAGGGGTGTTATGAGGGAAGCGGTGATCG | This work |
| R1-pBAMD1-2-SmR | GGGTCCGCAATTAATTAGACAAGGGTCTTATTTGCCGACTACCTTGGT | This work |
| F1-pBAMD1-2-GmR | TGCTTACATAAACAGTAATACAAGGGGTGTTATGTTACGCAGCAGCAACG | This work |
| R1-pBAMD1-2-GmR | GGGTCCGCAATTAATTAGACAAGGGTCTTAGGTGGCGGTACTTGGG | This work |

**Table S3. Oligonucleotides used for sequencing.** These were synthesized by Integrated DNA Technologies (IDT-DNA). Note that PSBLA can be used with any SEVA vector that contains the ApR gene bla.

| **Oligonucleotide** | **Sequence** | **Reference** |
| --- | --- | --- |
| PS1 | AGGGCGGCGGATTTGTCC | (Silva-Rocha et al., 2013) |
| PS2 | GCGGCAACCGAGCGTTC | (Silva-Rocha et al., 2013) |
| PS3 | GAACGCTCGGTTGCCGC | (Silva-Rocha et al., 2013) |
| PS4 | CCAGCCTCGCAGAGCAGG | (Silva-Rocha et al., 2013) |
| PS5 | CCCTGCTTCGGGGTCATT | (Silva-Rocha et al., 2013) |
| PS6 | GGACAAATCCGCCGCCCT | (Silva-Rocha et al., 2013) |
| PSBLA | ATGCTGAATGCTCATACTC | This work |

**Table S4. Oligonucleotides used for arbitrary PCR and cargo control.** These were synthesized by Integrated DNA Technologies (IDT-DNA).

| **Oligonucleotide** | **Sequence** | **Reference** |
| --- | --- | --- |
| ARB6 | GGCACGCGTCGACTAGTACNNNNNNNNNNACGCC | (Das et al., 2005) |
| ARB2 | GGCACGCGTCGACTAGTAC | (Das et al., 2005) |
| ME-O-Km-Ext-F | CGTCTGTTTCAGAAATATGGCAT | (Martínez-García et al., 2014) |
| ME-O-Km-Int-F | ATCTGATGCTGGATGAATTTTTC | (Martínez-García et al., 2014) |
| ME-O-Sm-Ext-F | CTTGGCCTCGCGCGCAGATCAG | (Martínez-García et al., 2014) |
| ME-O-Sm-Int-F | CACCAAGGTAGTCGGCAAAT | (Martínez-García et al., 2014) |
| ME-O-Gm-Ext-F | GCACTTTGATATCGACCCAAGT | (Martínez-García et al., 2014) |
| ME-O-Gm-Int-F | TCCCGGCCGCGGAGTTGTTCGG | (Martínez-García et al., 2014) |
| ME-O-Km-Ext-R | ATGCCATATTTCTGAAACAGACG | This work |
| ME-O-Sm-Ext-R | CTGATCTGCGCGCGAGGCCAAG | This work |
| ME-O-Gm-Ext-R | ACTTGGGTCGATATCAAAGTGC | This work |
| PSMCS | TTGTGTCTCAGGCCGCCT | This work |

**Table S5. Annotated sequencing results of *P. putida* insertion sites using delivery plasmids pBLAM1-2, pBLAM1-4 and pBLAM1-6.**

| **Delivery plasmid** | **Insertion location** | **Strand** | **Locus tag** |
| --- | --- | --- | --- |
| pBLAM1-2 | 3631884 | plus | PP_5590 |
| pBLAM1-2 | 6093056 | minus | PP_5345 |
| pBLAM1-2 | 5904595 | minus | PP_5177 |
| pBLAM1-2 | 5871011 | plus | PP_5145 |
| pBLAM1-2 | 5871011 | plus | PP_5145 |
| pBLAM1-2 | 5871011 | plus | PP_5145 |
| pBLAM1-2 | 5871002 | Plus | PP_5145 |
| pBLAM1-2 | 5871011 | plus | PP_5145 |
| pBLAM1-2 | 5871011 | plus | PP_5145 |
| pBLAM1-2 | 5722736 | minus | PP_5023 |
| pBLAM1-2 | 5633887 | minus | PP_4947 |
| pBLAM1-2 | 5314725 | minus | PP_4676 |
| pBLAM1-2 | 5296661 | minus | PP_4670 |
| pBLAM1-2 | 5188717 | plus | PP_4568 |
| pBLAM1-2 | 5089220 | minus | PP_4479 |
| pBLAM1-2 | 4570420 | plus | PP_4053 |
| pBLAM1-2 | 4533821 | plus | PP_4021 |
| pBLAM1-2 | 4465500 | plus | PP_3957 |
| pBLAM1-2 | 2437715 | minus | PP_2136 |
| pBLAM1-2 | 2094957 | minus | - |
| pBLAM1-4 | 1339995 | plus | PP_1164 |
| pBLAM1-4 | 100032 | plus | PP_0094 |
| pBLAM1-4 | 445725 | plus | PP_0368 |
| pBLAM1-4 | 5869492 | plus | Intergenic |
| pBLAM1-4 | 1536199 | minus | PP_1346 |
| pBLAM1-4 | 5191923 | plus | PP_4572 |
| pBLAM1-4 | 4877571 | plus | PP_4286 |
| pBLAM1-4 | 4670156 | minus | PP_4131 |
| pBLAM1-4 | 1001370 | minus | PP_0863 |
| pBLAM1-4 | 4664877 | minus | PP_4126 |
| pBLAM1-4 | 5466582 | plus | PP_4803 |
| pBLAM1-4 | 3464345 | minus | PP_3078 |
| pBLAM1-4 | 1461003 | minus | PP_1279 |
| pBLAM1-4 | 5092852 | plus | PP_4482 |
| pBLAM1-4 | 3981354 | plus | PP_3508 |
| pBLAM1-4 | 1337519 | minus | PP_1163 |
| pBLAM1-4 | 104083 | minus | PP_0099 |
| pBLAM1-4 | 1055398 | plus | PP_0913 |
| pBLAM1-4 | 4849229 | minus | PP_4264 |
| pBLAM1-4 | 1630245 | plus | PP_1430 |
| pBLAM1-6 | 781370 | minus | PP_0671 |
| pBLAM1-6 | 781549 | minus | PP_0671 |
| pBLAM1-6 | 156946 | minus | PP_0147 |
| pBLAM1-6 | 781503 | minus | PP_0671 |
| pBLAM1-6 | 781503 | minus | PP_0671 |
| pBLAM1-6 | 781519 | minus | PP_0671 |
| pBLAM1-6 | 781470 | minus | PP_0671 |
| pBLAM1-6 | 781460 | minus | PP_0671 |
| pBLAM1-6 | 781519 | minus | PP_0671 |
| pBLAM1-6 | 781519 | minus | PP_0671 |
| pBLAM1-6 | 781519 | minus | PP_0671 |
| pBLAM1-6 | 781517 | minus | PP_0671 |
| pBLAM1-6 | 781510 | minus | PP_0671 |
| pBLAM1-6 | 781539 | minus | PP_0671 |
| pBLAM1-6 | 781539 | minus | PP_0671 |
| pBLAM1-6 | 781558 | minus | PP_0671 |
| pBLAM1-6 | 781519 | minus | PP_0671 |
| pBLAM1-6 | 781509 | minus | PP_0671 |
| pBLAM1-6 | 781539 | minus | PP_0671 |
| pBLAM1-6 | 781539 | minus | PP_0671 |
